# Influence of methylsulfonylmethane on markers of exercise recovery and performance in healthy men: a pilot study

**DOI:** 10.1186/1550-2783-9-46

**Published:** 2012-09-27

**Authors:** Douglas S Kalman, Samantha Feldman, Andrew R Scheinberg, Diane R Krieger, Richard J Bloomer

**Affiliations:** 1 Miami Research Associates, Nutrition/Endocrinology Department, 6141 Sunset Drive Suite 301, Miami, FL, 33143, USA; 2 Department of Health and Sport Sciences, The University of Memphis, Cardiorespiratory/Metabolic Laboratory, 106 Roane Fieldhouse, Memphis, TN 38152, USA

**Keywords:** Methylsulfonylmethane, Exercise, Oxidative stress, Performance

## Abstract

**Background:**

Methylsulfonylmethane (MSM) has been reported to provide anti-inflammatory and antioxidant effects in both animal and man. Strenuous resistance exercise has the potential to induce both inflammation and oxidative stress. Using a pilot (proof of concept) study design, we determined the influence of MSM on markers of exercise recovery and performance in healthy men.

**Methods:**

Eight, healthy men (27.1 ± 6.9 yrs old) who were considered to be moderately exercise-trained (exercising <150 minutes per week) were randomly assigned to ingest MSM at either 1.5 grams per day or 3.0 grams per day for 30 days (28 days before and 2 days following exercise). Before and after the 28 day intervention period, subjects performed 18 sets of knee extension exercise in an attempt to induce muscle damage (and to be used partly as a measure of exercise performance). Sets 1–15 were performed at a predetermined weight for 10 repetitions each, while sets 16–18 were performed to muscular failure. Muscle soreness (using a 5-point Likert scale), fatigue (using the fatigue-inertia subset of the Profile of Mood States), blood antioxidant status (glutathione and Trolox Equivalent Antioxidant Capacity [TEAC]), and blood homocysteine were measured before and after exercise, pre and post intervention. Exercise performance (total work performed during sets 16–18 of knee extension testing) was also measured pre and post intervention.

**Results:**

Muscle soreness increased following exercise and a trend was noted for a reduction in muscle soreness with 3.0 grams versus 1.5 grams of MSM (p = 0.080), with a 1.0 point difference between dosages. Fatigue was slightly reduced with MSM (p = 0.073 with 3.0 grams; p = 0.087 for both dosages combined). TEAC increased significantly following exercise with 3.0 grams of MSM (p = 0.035), while homocysteine decreased following exercise for both dosages combined (p = 0.007). No significant effects were noted for glutathione or total work performed during knee extension testing (p > 0.05).

**Conclusion:**

MSM, especially when provided at 3.0 grams per day, may favorably influence selected markers of exercise recovery. More work is needed to extend these findings, in particular using a larger sample of subjects and the inclusion of additional markers of exercise recovery and performance.

## Background

Methylsulfonylmethane (MSM) is a naturally occurring nutrient composed of sulfur, oxygen and methyl groups [1]. In the presence of ozone and high-energy ultraviolet light, MSM (along with dimethyl sulfoxide [DMSO]) is formed from dimethyl sulfide, taken up into atmosphere, returned to the earth in rainfall, and taken into the root systems of plants. As such, MSM can be found in small quantities in a variety of foods [2], such as milk, fruits and vegetables (e.g., tomatoes, corn), coffee, and tea. While multiple health-related benefits are attributed to sulfur in general [3], and to MSM specifically—ranging from improved physical function [4] to a potential reduction in certain cancer risk [5], the proposed mechanisms of action for MSM appear related to both anti-inflammatory [6] and anti-oxidative activity [7].

MSM may inhibit the translocation of the p65 subunit of nuclear factor (NF)-kß to the nucleus [6], thus minimizing downstream events associated with local and systemic inflammation. Indeed, supplementation with MSM may minimize the expression of pro-inflammatory cytokines [8]. MSM has been reported to increase antioxidant defense (glutathione) [9], as well as decrease the actual production of reactive oxygen species (ROS) [7]. As with pro-inflammatory biomarkers, supplementation with MSM has resulted in a lowering of multiple oxidative stress biomarkers [10,11].

Collectively, these findings suggest that MSM might favorably influence exercise recovery, as both inflammation and oxidative stress may be involved in the etiology of exercise-induced muscle damage and associated symptoms [12]. Considering this and the excellent safety profile of MSM, we used a pilot (proof of concept) study design to determine the influence of MSM on markers of exercise recovery and performance in healthy men. At the time of study conception, we were unaware of any published trials focused on the use of MSM as a potential exercise recovery agent. We hypothesized that MSM would favorably influence our outcome measures (e.g., reduce muscle soreness, reduce muscle fatigue, increase antioxidant capacity), providing justification for further study of this ingredient using a larger scale, placebo controlled study design.

## Methods

### Subjects and screening

Eight healthy men (27.1 ± 6.9 yrs old) who were considered to be moderately exercise-trained (exercising <150 minutes per week) were recruited to participate in an open label (unblinded) pilot study. Eligibility was determined by completion of a health history form (Physical Activity Readiness Questionnaire [PAR-Q]) and physical examination. All subjects had experience performing resistance exercise, to ensure that the exercise protocol they were exposed to in the present design did not present a novel challenge. All subjects were instructed to maintain their pre-study exercise program throughout the course of the study, with the exception of refraining from exercise during the 24 hours prior to each test day and during the 48 hours following each test day. Subjects were nonsmokers, did not report any history of cardiovascular, metabolic, neurological, muscular, or orthopedic disorders that may have impacted their ability to participate in the study, and did not start the use of any new nutritional supplement or medication over the course of the study. However, subjects were allowed to continue using nutritional supplements and medications they had been using prior to beginning the study (e.g., multivitamins, acetaminophen), with the exception of the 24 hours prior to each test day and the 48 hours following each test day. Prior to participation, each subject was informed of all procedures, potential risks, and benefits associated with the study through both verbal and written form in accordance with the approved procedures of the Aspire Institutional Review Board for Human Subjects Research (La Mesa, CA; approval date of March 1, 2011). Subjects signed an informed consent form prior to being admitted into the study.

At the screening visits, the subjects’ height via stadiometer (Holtain Limited; Britain) and body mass via digital scale (Detecto; Webb City, MO) were measured and recorded. Body mass was obtained with subjects wearing only a gown and underwear. Heart rate and blood pressure (using subjects’ left arm) were recorded following a minimum of five minutes of quiet rest, while seated in a chair. A 12-lead electrocardiogram was obtained and analyzed for normality, to ensure subject suitability for participation. A blood sample was collected from subjects for routine assessment of clinical chemistry parameters (e.g., metabolic panel and complete blood count). Please see Table 1 for subject descriptive characteristics and Table 2 for blood parameters. During the initial laboratory visit, a 1-repetition maximum (1-RM) test for the knee extension exercise was also conducted using standard procedures, allowing 2–4 minutes between successive attempts. In addition, a familiarization trial of the exercise protocol was performed (one set of 10 repetitions performed at 30%, 45%, 60% and 70% 1-RM for a total of 40 repetitions).

**Table 1:** Characteristics of 8 healthy men assigned to MSM

**Variable**	**1.5 g/day (n = 4)**	**3.0 g/day (n = 4)**	**All Subjects**	**p-value**
Age (yrs)	31.5 ± 5.9	22.8 ± 4.9	27.1 ± 6.9	0.063
	33.5 (23.0 – 36.0)	21 (19.0 – 30.0)	26.5 (19.0 – 36.0)	
Height (cm)	175.5 ± 4.4	177.0 ± 2.2	176.3 ± 3.3	0.565
	175.0 (171.0 – 181.0)	176.5 (175.0 – 180.0)	176.5 (171.0 – 181.0)	
Weight (kg)	75.0 ± 5.3	75.0 ± 3.9	75.0 ± 4.3	0.988
	75.7 (68.0 – 80.8)	73.3 (72.4 – 80.8)	74.4 (68.0 – 80.8)	
BMI (kg·m^-2^)	24.4 ± 1.6	23.9 ± 1.5	24.2 ± 1.4	0.703
	24.5 (22.8 – 25.8)	23.9 (22.3 – 25.8)	23.9 (22.3 – 25.8)	
SBP (mm Hg)	118.0 ± 2.9	110.0 ± 14.9	114.0 ± 10.8	0.772
	118.5 (114.0 – 121.0)	115.0 (89.0 – 121.0)	118.5 (89.0 – 121.0)	
DBP (mm Hg)	75.5 ± 2.1	73.0 ± 8.2	74.3 ± 5.7	0.576
	75.5 (73.0 – 78.0)	74.5 (62.0 – 81.0)	75.5 (62.0 – 81.0)	
HR (bpm)	58.3 ± 4.9	66.3 ± 6.7	62.3 ± 6.9	0.103
	58.0 (54.0 – 63.0)	64 (61.0 – 76.0)	62.5 (54.0 – 76.0)	
QTcB (msec)	383.5 ± 7.6	376.8 ± 15.6	380.1 ± 11.9	0.470
	383.7 (374.0 – 392.5)	374.4 (360.3 – 398.0)	379.3 (360.3 – 398.0)	

Data are mean ± SD (top row); median and (range) provided in bottom row.

**Table 2:** Metabolic panel and blood counts of 8 healthy men assigned to MSM

**Variable**	**1.5 g/day (n = 4)**	**3.0 g/day (n = 4)**	**All Subjects**	**p-value**
Glucose (mg·dL^-1^)	85.3 ± 2.6	96.3 ± 7.1	90.8 ± 7.7	0.028
	85.0 (83.0 – 88.0)	94.5 (90.0 – 106.0)	89.0 (83.0 – 106.0)	
BUN (mg·dL^-1^)	15.8 ± 4.8	12.8 ± 2.6	14.3 ± 3.9	0.314
	16.0 (10.0 – 21.0)	13.5 (9.0 – 15.0)	14.0 (9.0 – 21.0)	
Creatinine (mg·dL^-1^)	1.0 ± 0.1	0.9 ± 0.1	1.0 ± 0.1	0.561
	1.0 (0.8 – 1.0)	0.9 (0.8 – 1.0)	1.0 (0.8 – 1.0)	
AP (Units·L^-1^)	73.5 ± 25.0	85.0 ± 23.4	79.3 ± 23.2	0.527
	71.0 (47.0 – 105.0)	78.0 (66.0 – 118.0)	75.5 (47.0 – 118.0)	
AST (Units·L^-1^)	19.8 ± 4.9	16.0 ± 2.4	17.9 ± 4.1	0.222
	19.5 (14.0 – 26.0)	16.5 (13.0 – 18.0)	18.0 (13.0 – 26.0)	
ALT (Units·L^-1^)	21.3 ± 10.9	20.0 ± 6.7	20.6 ± 8.4	0.851
	17.0 (14.0 – 37.0)	21.0 (11.0 – 27.0)	20.0 (11.0 – 37.0)	
WBC (thousand·μL^-1^)	5.60 ± 1.49	7.10 ± 1.79	6.35 ± 1.72	0.245
	5.9 (3.6 – 7.1)	7.1 (5.0 – 9.3)	6.4 (3.6 – 9.3)	
RBC (million·μL^-1^)	5.2 ± 0.3	5.4 ± 0.2	5.3 ± 0.3	0.255
	5.1 (4.9 – 5.7)	5.5 (5.2 – 5.6)	5.3 (4.9 – 5.7)	
Hemoglobin (g·dL^-1^)	14.9 ± 0.4	15.7 ± 0.9	15.3 ± 0.8	0.172
	14.9 (14.5 – 15.3)	15.8 (14.6 – 16.6)	15.2 (14.5 – 16.6)	
Hematocrit (%)	48.2 ± 2.0	49.9 ± 2.3	49.0 ± 2.2	0.313
	48.0 (46.4 – 50.3)	50.2 (47.2 – 51.8)	49.1 (46.4 – 51.8)	

Data are mean ± SD (top row); median and (range) provided in bottom row.

### Supplementation

Subjects were randomly assigned (via a Block-2 randomization scheme) to ingest MSM at either 1.5 grams per day (n = 4) or 3.0 grams per day (n = 4) for 28 days prior to performing the exercise test, in addition to the two days following the exercise test (i.e., the recovery period). Subjects were instructed to begin the supplementation two days following the initial exercise test session, once data collection for the recovery period was completed. Please see Table 3 for a depiction of the study variables and timeline for measurement. The MSM (OptiMSM®) was provided by Bergstrom Nutrition (Vancouver, WA) and was produced under Good Manufacturing Practice. Capsules contained 750 mg of MSM and subjects ingested two or four capsules daily (divided into a morning and night dosage) in order to provide the dosage of 1.5 or 3.0 grams, respectively. No blood measurement of MSM before and after supplementation was included in this study. Hence, capsule counts upon bottle return allowed for the sole calculation of compliance to treatment.

**Table 3:** Study timeline and outcome measures

**Time →**	Pre/Post MSM*	**Pre/Post MSM**	**Pre/Post MSM**	**Pre/Post MSM**
**Variable ↓**	***Pre exercise***	***Immediately post-exercise***	***2 hours Post-exercise***	***48 hours Post-exercise***
Muscle Soreness	X		X	X
Fatigue Questionnaire	X		X	X
Exercise Performance		X		
Blood Homocysteine	X	X	X	
Blood TEAC	X	X	X	
Blood Glutathione	X	X	X	

* Pre/Post MSM refers to Pre intervention (prior to use of MSM; test visit 1) and Post Intervention (after supplementation with MSM; test visit 2).

TEAC: Trolox Equivalent Antioxidant Capacity.

### Physical activity and dietary intake

Subjects were instructed to maintain their normal physical activity throughout the study period, with the exception of refraining from strenuous physical activity during the 24 hours prior to each test day and during the 48 hours following each test day. They were also given specific instructions regarding abstinence from alcohol, medication, and dietary supplement consumption during the 24 hours immediately before the test days and during the 48 hours following each test day. Dietary intake was to be maintained as usual through the study period, with the exception of reporting to the lab in a fasted state on each of the two test days (no food, caffeine, or calorie containing beverages allowed after midnight). No food records were maintained in this study, which may be considered a limitation of this work.

### Exercise test days

On each of the two exercise test days, subjects reported to the lab in the morning following an overnight fast. However, subjects were instructed to consume water liberally up to the time they reported to the lab for testing. Adherence to study instructions was confirmed with all subjects on each day of testing by use of a dichotomous questionnaire. Specifically, on the day of testing, we used an in-lab questionnaire asking subjects if they consumed any food since midnight the night before, or any alcohol, caffeine, or nutritional supplements during the prior 24 hours. We also used phone questionnaires during the study period asking subjects if they exercised since the last study visit, used any vitamin and/or mineral supplements since the last study visit, or taken acetaminophen since the last study visit.

For testing days, the time of day for each subject was matched for the subsequent test day. Following all baseline measurements and approximately 45 minutes prior to the start of the knee extension exercise protocol, subjects were provided with a standardized breakfast consisting of a bagel, one tablespoon of low fat cream cheese, 8 ounces of orange juice, and water *ad libitum*. On the test days, subjects took their assigned MSM dose immediately prior to the standardized breakfast.

For the exercise test, subjects performed a total of 18 sets of knee extension exercise using a plate-loaded machine (Key Fitness Products, LP; Garland, TX). Sets 1–15 were performed at a predetermined weight for 10 repetitions each, while sets 16–18 were performed to muscular failure. Specifically, subjects performed 5 sets of 10 repetitions at 30% 1-RM for a total of 50 repetitions, followed by a 3 minute rest. Subjects then performed 5 sets of 10 repetitions at 45% 1-RM for a total of 50 repetitions, followed by a 3 minute rest. Subjects then performed 5 sets of 10 repetitions at 60% 1-RM for a total of 50 repetitions, followed by a 20 minute rest (to allow muscle recovery and re-hydration as needed—water allowed *ad libitum*).

Following these initial 15 sets in which the repetition number was standardized, subjects performed 3 sets of repetitions to failure at 70% 1-RM, with 3 minutes of rest between each set. The total number of repetitions performed was counted and used as an indicator of total work performed (reps x load = volume load). Before and following the completion of the exercise test, outcome measures were assessed as indicated below. It should be noted that the exercise protocol used in this study is similar in volume as other exercise protocols used to induce muscle fatigue. However, to our knowledge, this exact protocol has not been used in other published work focused on muscle injury and oxidative stress, but was developed based on general resistance exercise guidelines presented in published form [13]. In hindsight, although the protocol was of similar volume as those used in past studies of muscle injury and oxidative stress, the overall intensity of work may not have been great enough to induce adequate muscle damage and oxidative stress, as the ideal protocol may have included not only high volume exercise but also high force exercise (i.e., pure eccentric muscle actions), which are known to induce significant muscle trauma [14].

### Outcome measures

All outcome measures were determined both pre and post intervention (i.e., before and after intake of MSM). As described in past research [15,16], muscle soreness was determined using a visual analog scale: In this study we used a 5-point Likert scale (0 = no soreness at all; 4 = very sore). The muscle soreness questionnaire was administered before exercise and 2 and 48 hours following the knee extension protocol with subjects reporting the level of soreness in their legs (quadriceps) “right now.” In addition to muscle soreness, fatigue was determined using a distinct questionnaire—the fatigue-inertia subset of the Profile of Mood States [17,18], which includes a 5-point Likert scale (0 = not at all, 1 = a little, 2 = moderately, 3 = quite a bit, 4 = extremely). The fatigue questionnaire was also administered before exercise and 2 and 48 hours post-exercise with subjects reporting their level of fatigue “right now.” Although some overlap may be present in individuals’ view and rating of soreness and fatigue, our questionnaires were distinct and clearly represented either soreness or fatigue, both of which were rated by subjects. Exercise performance during the final three sets of knee extension was determined based on total volume load (reps x load).

Heart rate and blood pressure were measured, and venous blood was collected from subjects before exercise, immediately post-exercise, and two hours post-exercise. Blood from tubes containing EDTA was used for total (TGSH) and oxidized (GSSG) glutathione analysis. Specifically, whole blood was immediately deproteinated by mixing with fresh, ice cold 5% metaphosphoric acid. The mixture was vortexed and then centrifuged at 10,000 g for 5 minutes. The supernatant was removed and immediately stored at −70°C. The remaining whole blood from EDTA tubes was then centrifuged at 1500 g at 4°C for 15 min to obtain plasma. Collection tubes containing no additive were allowed to clot at room temperature for 30 minutes and then centrifuged at 1500 g at 4°C for 15 min to obtain serum. Blood aliquots were stored at −70°C until assayed, except for homocysteine which was analyzed in fresh plasma using a competitive immunoassay format (Tri-State Clinical Laboratory Services, LLC, Cincinnati, OH).

Antioxidant capacity was analyzed in serum using the Trolox-equivalent antioxidant capacity (TEAC) assay using procedures outlined by the reagent provider (Sigma Chemical, St. Louis, MO). The coefficient of variation (CV) was 5.2%. For the analysis of glutathione, whole blood was first deproteinated using 5% metaphosphoric acid, as indicated above. The supernatant was then used to separately assay for TGSH and GSSG, and reduced glutathione (GSH) was calculated mathematically. For analysis of GSSG, supernatants were first neutralized with NaOH, and then 4-vinylpyridine was mixed with the supernatant and incubated at room temperature for one hour in order to derivatize GSH. Assays for glutathione were performed using commercially available reagents (Northwest Life Science Specialties, Vancouver, WA). The CV for TGSH and GSSG was 3.2% and 4.9%, respectively.

### Statistical analysis

This was a small exploratory pilot/proof of concept study, and it was not expected that significant changes over time, or significant differences between treatment groups, would be observed unless the differences were very large. Therefore the efficacy analysis described below should be considered only a formality; the main purpose of this study was to generate a general sense of the response of subjects to the two doses of MSM and to obtain estimates of endpoint variability and other parameters that could be used to inform the design of a larger, more definitive study, if one were to be carried out. The *acute* changes over the course of the testing visit, and the *long-term* changes over the course of a one-month MSM administration period, were tested for significance *within* each group, and *between* the two groups. Each outcome measure was tested using an analysis of covariance (ANCOVA), with the value of the variable at the end of the study being the dependent variable, the dose being the main factor, and the value of the variable at baseline being the covariate. The coefficient of the product (relative to dose) and its standard error of estimate were calculated from the ANCOVA. Significant product efficacy was inferred if this coefficient was significantly different from zero. Analyses were performed using “*R*” statistical software (version 2.13.1; *R* Foundation for Statistical Computing). Statistical significance was set at p ≤ 0.05. The data are presented within the text, Tables, and Figures as mean ± SD.

## Results

### Overview and adverse effects

All subjects successfully completed all aspects of this study. Compliance to capsule intake was 99.9 ± 7.6, considering all subjects. No serious adverse events were observed during this study. However, one subject in the 1.5 grams/day MSM group reported mild nausea during his last visit. Heart rate and blood pressure responded as expected to acute exercise (these variables increased slightly and returned to baseline rapidly) and were not differently influenced by either dosage of MSM (p > 0.05).

### Recovery and performance data

**Figure 1:**
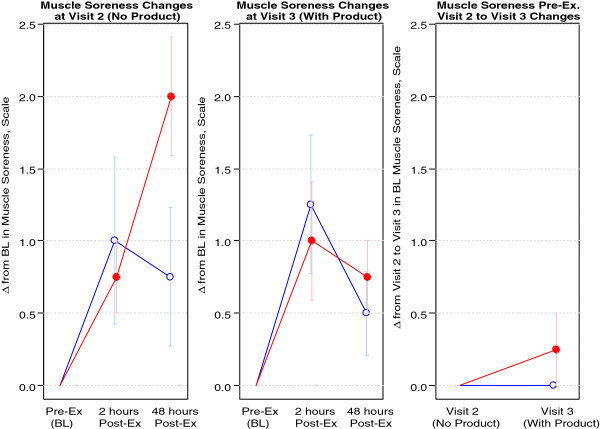
**Muscle soreness of 8 healthy men assigned to MSM.** Blue Open Circle = 1.5 grams/day; Red Filled Circle = 3.0 grams/day. Data are presented as change from baseline (Δ from BL) on y-axis; Visit 2 is pre intervention (prior to MSM supplementation), Visit 3 is post intervention (following MSM supplementation); Visit 1 included the screening visit. Note: There were statistically significant increases in muscle soreness with and without MSM at the two hour post-exercise time (p= 0.021 and p=0.007, respectively); The 1.5 grams/day group experienced a 0.5 point greater reduction in muscle soreness during Visit 3 (post intervention) as compared to Visit 2 (pre intervention), and the 3.0 grams/day group experienced a 1.5 point greater reduction in soreness during Visit 3 as compared to Visit 2. This 1.0 point difference in baseline-adjusted muscle soreness from two hours post-exercise to 48 hours post-exercise approached statistical significance (p=0.080), suggesting a dose-related improvement.

Regarding muscle soreness, the 1.5 grams/day group experienced a 0.5 point greater reduction in muscle soreness during the post intervention visit as compared to pre intervention, and the 3.0 grams/day group experienced a 1.5 point greater reduction in soreness during the post intervention visit as compared to pre intervention. This 1.0 point difference in baseline-adjusted muscle soreness from two hours post-exercise to 48 hours post-exercise approached statistical significance (p = 0.080), suggesting a dose-related improvement. The Cohen's D value for the outcome of muscle soreness was 0.28 and the Pearson's r value (effect size) was 0.14. Muscle soreness data are presented in Figure 1.

**Figure 2:**
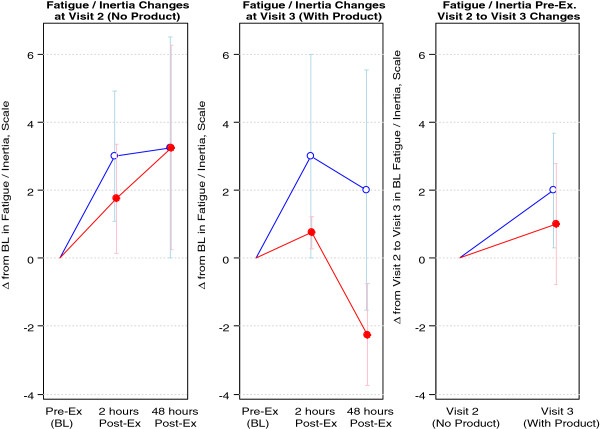
**Fatigue of 8 healthy men assigned to MSM.** Blue Open Circle = 1.5 grams/day; Red Filled Circle = 3.0 grams/day. Data are presented as change from baseline (Δ from BL) on y-axis; Visit 2 is pre intervention (prior to MSM supplementation), Visit 3 is post intervention (following MSM supplementation); Visit 1 included the screening visit. Note: All subjects experienced an increase in fatigue that trended towards significance two hours post-exercise at Visit 2 (pre intervention; p=0.084), whereas there was no trend at Visit 3 (post intervention; p=0.181); At Visit 2, subjects’ fatigue scores increased between two and 48 hours post-exercise, but not significantly (p=0.47), whereas at Visit 3, subjects fatigue scores decreased between two and 48 hours post-exercise, but not significantly (p=0.336); the difference in these changes between Visits 2 and 3 trended toward statistical significance (for the 3.0 grams/day group [p=0.073] and for all subjects [p=0.087]).

Regarding fatigue, all subjects experienced an increase in fatigue that trended towards significance two hours post-exercise at the pre intervention visit (p = 0.084), whereas there was no trend at the post intervention visit (p = 0.181). At the pre intervention visit, subjects’ fatigue scores increased between two and 48 hours post-exercise, but not significantly (p = 0.470), whereas post intervention, subjects fatigue scores decreased between two and 48 hours post-exercise, but not significantly (p = 0.336). The difference in these changes between pre and post intervention trended toward statistical significance (for the 3.0 grams/day group [p = 0.073] and for all subjects [p = 0.087]). Fatigue data are presented in Figure 2.

There were no differences in the total work performed by subjects during the pre intervention (7,901 ± 3,226 kg) and post intervention (6,900 ± 2,029 kg) visits when pooling all subjects (p > 0.05). Nor was any difference noted when looking at the 1.5 gram (pre: 7,161 ± 2,511 kg; post: 6,644 ± 1,371 kg) and 3.0 gram (pre: 8,642 ± 4,064 kg; post: 7,155 ± 2,748 kg) groups independently (p > 0.05).

**Figure 3:**
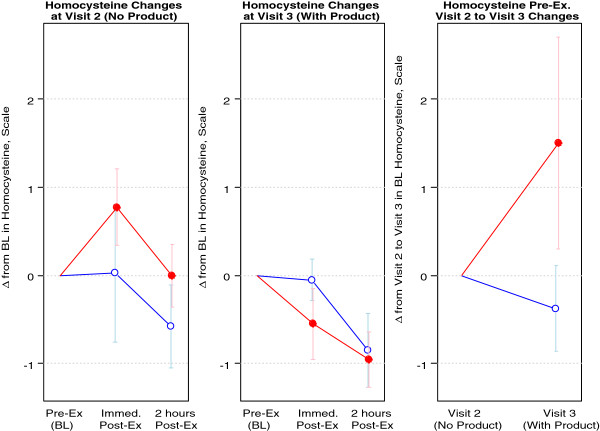
**Blood homocysteine of 8 healthy men assigned to MSM.** Blue Open Circle = 1.5 grams/day; Red Filled Circle = 3.0 grams/day. Data are presented as change from baseline (Δ from BL) on y-axis; Visit 2 is pre intervention (prior to MSM supplementation), Visit 3 is post intervention (following MSM supplementation); Visit 1 included the screening visit. Note: At Visit 2 (pre intervention), homocysteine levels were either unchanged or increased slightly immediately post-exercise, whereas at Visit 3 (post intervention), homocysteine levels decreased significantly in all subjects post-exercise (p= 0.007) and trended towards significance in the 3.0 grams/day group (p=0.056).

Regarding homocysteine, during the pre intervention visit, levels were either unchanged or increased slightly immediately post-exercise. Post intervention, homocysteine levels decreased significantly in all subjects post-exercise (p = 0.007) and trended towards significance in the 3.0 grams/day group (p = 0.056). Homocysteine data are presented in Figure 3.

**Figure 4:**
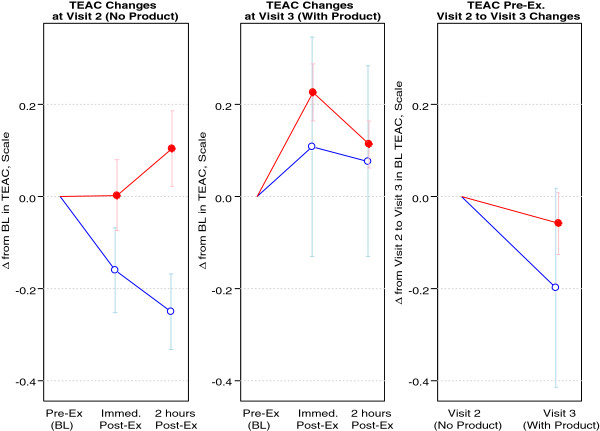
**Blood TEAC of 8 healthy men assigned to MSM.** Blue Open Circle = 1.5 grams/day; Red Filled Circle = 3.0 grams/day. Data are presented as change from baseline (Δ from BL) on y-axis; Visit 2 is pre intervention (prior to MSM supplementation), Visit 3 is post intervention (following MSM supplementation); Visit 1 included the screening visit. Note: There was a statistically significant increase in TEAC immediately post-exercise at Visit 3 (post intervention) for the 3.0 grams/day group (p=0.035). TEAC: Trolox Equivalent Antioxidant Capacity.

Regarding antioxidant capacity as measured by TEAC, there was a statistically significant increase immediately post-exercise for the 3.0 grams/day group (p = 0.035) at the post intervention test visit. TEAC data are presented in Figure 4. Glutathione status (total, oxidized, and reduced) was unaffected by exercise or MSM supplementation (p > 0.05; data not shown).

## Discussion

Findings from the present investigation indicate that MSM supplementation in healthy, moderately exercise-trained men may favorably influence selected markers of exercise recovery. This effect appeared to be greater with a daily dosage of 3.0 grams of MSM than a daily dosage of 1.5 grams. Although this study included a very small sample of subjects, which makes it difficult to confidently discuss the overall meaning of our findings, our data provide initial evidence that MSM may have efficacy in regards to influencing certain markers of exercise recovery. Further studies are needed, inclusive of a larger sample size (~15-20 subjects per group, if not larger), a placebo control group, and additional markers of exercise recovery and performance. In such future studies, analysis of blood MSM concentrations pre and post intervention, as opposed to simple capsule counts as done in the present design, would prove valuable as an indication of supplement compliance (as well as to provide information related to supplement absorption, etc.).

This is the first trial to note an impact of MSM on blood TEAC, suggesting increased antioxidant activity. This marker, like other “global” markers of antioxidant status (e.g., ORAC, FRAP, TRAP) provides a general measure of the sum total of antioxidants within blood and other tissues [19]. While the observed increase in TEAC may indeed have relevance, future studies focused on MSM should ideally include additional markers of antioxidant activity, as well as markers of oxidative stress.

While TEAC was noted to be higher post-exercise with MSM, we did not observe the same finding for blood glutathione, which appeared unaffected by exercise or supplementation with MSM. Our results for glutathione oppose those of DiSilvestro et al. who noted an increase of 78% in *liver* glutathione when studying male mice ingesting MSM in drinking water for 5 weeks [9]. The present study, however, was quite different in design. For example, it involved human intake of MSM, glutathione measured in whole blood, and the inclusion of a physical stressor (i.e., 18 sets of knee extension exercise). These differences may be responsible for the discrepancies in findings.

As we believe that TEAC does in fact represent an increase in antioxidant defense (independent of glutathione), it is possible that this increase may have attenuated the commonly observed rise in ROS during and following exercise [20], resulting in attenuation of exercise-induced oxidative stress. While oxidative stress biomarkers were not included in the present design, extant literature indicates a potential for this effect. For example, we know of at least one animal study [11] and one human study [10] that has focused on the role of MSM to attenuate exercise-induced oxidative stress. Marañon and colleagues studied competitive jumping horses receiving either a standard control diet, a MSM diet (8 mg/kg MSM), or a combined MSM + vitamin C diet (8 mg/kg MSM + 5 mg/kg vitamin C) for a period leading up to competition [11]. Blood was collected before and within 15 minutes following competition and analyzed for a variety of oxidative stress markers. The competitive exercise resulted in noted increases in lipid peroxidation, nitric oxide metabolites, and carbon monoxide, with decreases in reduced glutathione and antioxidant enzyme activity. Supplementation with MSM significantly attenuated the observed changes due to competition, with a more pronounced effect noted with MSM + vitamin C treatment. Moreover, in a recently published human study [10], MSM supplementation at 50 mg/kg was provided to untrained healthy men for 10 days prior to performing a 14 km run. Blood was collected before and at times through 48 hours of exercise recovery and analyzed for lipid, protein, and glutathione oxidation. As expected, acute exercise resulted in an increase in oxidative stress; however, this increase was blunted significantly with MSM supplementation as compared to placebo. Collectively, the results of Marañon et al. [11] and Nakhostin-Roohi et al. [10] provide initial evidence that prophylactic intake of MSM prior to exercise may alleviate the oxidative stress that is often observed following strenuous bouts of exercise, in particular in those who are not accustomed to the stress of exercise [20]. Although ROS have been linked to potential problems in muscle integrity and the generation of muscle force [21], the above studies did not include any measure of physical performance in the design. This is certainly a limitation and such measures should be considered in future studies investigating the impact of MSM on exercise recovery.

Aside from measures of antioxidant status (TEAC and glutathione), we included the measure of homocysteine in the current design. Homocysteine is a non-protein amino acid, with elevated levels in circulation thought to be associated with an increased risk of cardiovascular disease; although recent evidence questions this association [22]. A study by Kim et al. reported a statistically significant lowering of homocysteine (8.0 to 7.2 μmol·L^-1^) in a sample of knee osteoarthritis patients following intake of MSM at a dosage of 6 grams per day for 12 weeks [4]. Data from the present investigation somewhat corroborate the work of Kim and colleagues, as we noted a lowering of homocysteine during the post-exercise period after subjects were supplemented with MSM for four weeks (Figure 3). The mechanism of action of this effect may be linked to methyl group donation by MSM, in much the same way as done by B-vitamins [23].

From a practical standpoint, we measured muscle performance by calculating total work performed during the final 3 sets of knee extension exercise. Despite some positive findings for our non-exercise performance measures, we failed to note any difference in exercise performance between pre and post intervention. In fact, values were actually lower following MSM supplementation as compared to before supplementation. We have no explanation for these findings other than recognizing our small sample size and the potential for day-to-day variance in knee extension “muscle endurance” performance, as has been noted for isokinetic testing [24]. Also noteworthy, motivation is paramount when asking subjects to perform repetitions to exhaustion.

In retrospect we believe that our chosen protocol may not have been ideal to discern performance differences between groups and across time. Although subjects performed a total of 18 sets of knee extension exercise, the first 15 sets were standardized in terms of repetition number. Hence, subjects were only provided a total of 3 performance sets (16–18) to generate usable data for performance comparison. Future work may include a different exercise protocol, with the possible addition of isometric and dynamic force, as well as power data as done previously [25], in addition to actual volume load (reps x load). This would provide for a more complete assessment of muscle performance—as well as greater potential for observed differences in muscle soreness and oxidative stress related parameters. Moreover, the “damaging” exercise protocol may be altered to include a more robust model for inducing damage (e.g., pure eccentric loading using 1-RM values that are far greater than those used in the present design) [16].

In addition to performance, we used two distinct questionnaires to determine the extent of either muscle soreness or fatigue, before and following exercise, both pre and post intervention. Although preliminary, MSM did provide some evidence of effect at attenuating both muscle soreness and fatigue (Figures 1 and 2, respectively). As with other measures, additional larger scale studies are needed to corroborate these findings. If future work agrees with these initial findings, MSM may serve a useful purpose in enhancing post-exercise recovery.

## Conclusion

Our data indicate that supplementation with MSM, specifically at a daily dosage of 3.0 grams, may favorably influence selected markers of exercise recovery. In particular, to our knowledge, this was the first study to observe an effect of MSM on antioxidant capacity, as measured by blood TEAC. While this study was small in scope, it is suggested that more research be done to extend these findings. Specifically, future studies should include a larger sample size, a placebo group for comparison, the inclusion of additional markers of recovery and exercise performance (e.g., force and power), as well as the consideration for a more strenuous “muscle damaging” protocol and a longer time course of post-exercise assessment (e.g., 4–7 days). Such work may help to more fully elucidate the role of MSM in exercise recovery.

## Competing interests

Financial support for this work was provided by TandemRain Innovations (Vancouver, WA). RJB has received research funding or has acted as a consultant to nutraceutical and dietary supplement companies.

## Authors' contributions

DSK, SF, ARS, and DRK were responsible for the study design, coordination of the study, and oversight of data collection and analysis. RJB assisted in manuscript preparation. All authors read and approved of the final manuscript.
